# 

**Published:** 1881-04

**Authors:** 


CONTENTS.

| | PAGE. |
|---|---|
| Causes of Diseases | 119 |
| Moderation in all Things | 120 |
| New Treatment of Diphtheria | 122 |
| A Great River in Alaska | 123 |
| Suggestion to Manager Abbey | 124 |
| Punctuality | 127 |
| Queer Industries in New York | 128 |
| A Reflection (poetry) | 128 |
| Cold Feet | 129 |
| Living in Italy | 130 |
| Heart Affections | 131 |
| Sprains | 132 |
| Outdoor Safety | 133 |
| Habit | 134 |
| Animal Ailments | 135 |
| Health | 139 |
| Coughing | 140 |
| Ice Water | 141 |
| Regimen | 142 |
| Sleeping | 143 |
| Small-Pox | 146 |
| The Foster-Brothers | 147 |
| The One Leaf (poetry) | 155 |
| Uncle Esek's Wisdom | 155 |

